# Seasonal influenza vaccine awareness and factors affecting vaccination in Turkish Society

**DOI:** 10.12669/pjms.38.4.4915

**Published:** 2022

**Authors:** Olgun Goktas, Fatma Ezgi Can, Burkay Yakar, Ilker Ercan, Emin Halis Akalin

**Affiliations:** 1 Dr. Olgun Goktas Associate Professor, Uludag University Family Health Center, Nilufer, Bursa, Turkey; 2 Dr. Fatma Ezgi Can Department of Biostatistics, Faculty of Medicine, Izmir K**â**tip Celebi University, Izmir, Turkey; 3 Dr. Burkay Yakar Associate Professor, Department of Family Medicine, Faculty of Medicine, Firat University, Elazig, Turkey; 4 Prof. Dr. Ilker Ercan Department of Biostatistics, Faculty of Medicine, Bursa Uludag University, Bursa, Turkey; 5 Prof. Dr. Emin Halis Akalin Department of Infectious Diseases and Clinical Microbiology, Faculty of Medicine, Bursa Uludag University, Bursa, Turkey

**Keywords:** Family medicine, General practice, Immunization, Seasonal influenza, Vaccine

## Abstract

**Objective:**

Influenza vaccine reduces the burden of seasonal influenza and related complications. Potential vaccination barriers need to be identified to raise awareness and increase acceptance. We aimed to investigate the rates of seasonal influenza vaccination and the knowledge, opinions, and behaviours prevalent in Turkish society.

**Methods:**

The study among seven regions in Turkey was conducted from October-November 2018 in 28 family health centres, using a cross-sectional, descriptive design. The knowledge, opinions, and behaviours of participants regarding the influenza vaccine were obtained by family physicians through face-to-face interviews with participants.

**Results:**

A total of 3,492 people aged 10-97 years age range (median: 50 years) were included in the study. Over half of the participants (59.9%, *n* = 2093) were female. It was found that the percentage of participants who never received the influenza vaccine was 78.4%; only 13.4% were occasionally vaccinated, and 8.1% received regular annual vaccination. Influenza vaccination rates were higher in married people (*p* < 0.001), women (*p* = 0.005), patients with chronic lung and cardiovascular disease (*p* < 0.001), those over 65 years /nursing home residents (*p* < 0.001). Awareness of the vaccine’s benefit was higher in the group at high risk of influenza (*p* < 0.001).

**Conclusion:**

The rate of regular vaccination against influenza every year was insufficient, at 8.1%. Individuals’ insensitivity, insufficient knowledge, and attitudes toward influenza vaccination is a serious health problem for Turkish society. Barriers to influenza vaccination can be reduced by good communication between family physicians and their patients.

## INTRODUCTION

Seasonal influenza is an important cause of mortality and causes a significant disease burden worldwide, leading to serious side-effects and complications that affect quality of life.^1^-^5^

Seasonal influenza vaccines are the most important way to reduce the disease. Regular annual vaccination against influenza decreases mortality and morbidity and decreases health expenditures, especially in older adult and at-risk patients.^6^,^7^ In this context, The Advisory Committee on Immunization Practices at the US Centres for Disease Control and Prevention (CDC) recommends an influenza vaccination once per year for all individuals older than six months, with specific exceptions.^8^ Both pneumococcal and influenza vaccines are strongly recommended for all individuals over the age of 65 who are at a higher risk for these conditions.^9^

Although the negative effect of influenza infections can be reduced with vaccination, vaccination rates are lower than deemed desirable. In Europe, influenza vaccination rates on average are 45%; if this rate were increased to 75%, the public health problems mentioned above could be reduced rapidly.^7^ In a Turkish study among participants over 65 years old the vaccination rate against influenza was reported to be only 33.9%.^10^ Determining obstacles to sufficient vaccination rates may contribute to improving immunization rates. In the literature, socio-demographic factors, insufficient knowledge, individual risk-benefit perceptions, and political, geographical, and financial factors have been reported to influence vaccination rates.^11^,^12^

Vaccination rates can be increased by raising the public’s knowledge and awareness of vaccines. Because family physicians are in close contact with individuals, it is important that they raise awareness about seasonal influenza vaccines among patients and offer preventive care.

In this context, we investigated seasonal influenza vaccination rates and patients’ knowledge, attitudes, and behaviours regarding vaccines. The current study data can contribute to a better understanding of vaccines in a Turkish context, and provide insights into Turkish knowledge, attitudes, and behavioural changes regarding vaccinations.

## METHODS

This cross-sectional, descriptive study was conducted at Family Health Centres in different regions of Turkey between October 2018 and November 2018. Turkey’s population is approximately 80 million people, divided among seven geographical regions. The study was conducted in 28 family health centres in nine cities, four from each geographical region, through 112 family physicians. Family physicians in family health centres from seven regions agreed to participate in the study. The sample size was calculated based on the 20.0% uptake of the influenza vaccine found among participants of a similar study by Sagor et al. The minimum sample size was calculated as n = 2389 at the d = 0.02 margin of error and α = 0.01 significance level.^13^ All individuals who randomly applied to Family Health centres were invited to the study in order of their arrival. Patients who were eligible for the inclusion criteria agreed to participate in the study. The family physicians were asked to provide the questionnaire to at least 30 individuals. All of the participants who were offered a questionnaire shared their opinions about the vaccine. Those without contraindications for vaccination and aged over three years were included in the study. Participants with physical disabilities, mental and neurological diseases, and contraindications for influenza vaccine administration were excluded from the study. After obtaining written consent from patients who visited the family health centre for any reason, a questionnaire was administered by the family physician. The answers were recorded synchronously via an online web link provided by Vademecum medication Guide Company.

The study was performed after receiving the approval of the Clinical Ethics Committee of Bursa Uludag University (Reference no: 2018-17/10, dated: 16.10.2018) and in accordance with the Declaration of Helsinki.

The study data were obtained via a questionnaire consisting of 16 ‘yes-no’ questions. The questionnaire addressed the sociodemographic characteristics of the participants, their vaccination status, risk factors for influenza infection, and their knowledge, attitudes, and behaviours regarding vaccines. The questionnaire was given to patients who visited family medicine centres through face-to-face interviews by family physicians. The average response time for the questionnaire was 15 minutes. The responses were recorded directly through the web link provided by the Vademecum drug guide company during the interview. The study was conducted anonymously and personal data such as name, surname, and address information were not requested from the participants. Written informed consent was obtained from all participants, who volunteered to participate in the study before the questionnaire was provided.

It is known that some sociodemographic characteristics are related to vaccination acceptance. Considering the previous literature data, the current study was questioned the relationship between vaccine acceptance and sociodemographic characteristics such as age, gender, educational status, marital status, social security, smoking and history of influenza.^14^

Participants were divided into three groups according to their vaccination characteristics. Group-1 was defined as never vaccinated, Group-2 as regularly vaccinated (annually) (those who regularly get the influenza vaccine in September-October every year), and Group-3 as irregularly vaccinated.

Participant risk factors for influenza infection included: (1) being over the age of 65, (2) staying at home for older adults or those in a nursing home, (3) being pregnant, (4) having chronic pulmonary or cardiovascular disease, (5) having chronic metabolic disease or chronic renal dysfunction or hemoglobinopathy, (6) People with a weakened immune system due to disease (such as people with HIV or AIDS, or some cancers such as leukaemia) or medications (such as those receiving chemotherapy or radiation treatment for cancer, or persons with chronic conditions requiring chronic corticosteroids or other drugs that suppress the immune system or people younger than 19 years old on long-term aspirin- or salicylate-containing medications.). The risk status of the participants was obtained by answering yes-no questions. Participants with any risk factors mentioned above were defined as the at-risk group for influenza.^8^

The knowledge, opinions and behaviours of the participants were questioned using yes-no questions. We asked participants; 1) is the vaccine useful? 2) the cost of the vaccine, 3) the harms of the vaccine, 4) the side effects of the vaccine, 5) who are the priority groups for vaccination, 6) if the vaccine is free of charge, would you like to be vaccinated?

Data were analysed using the SPSS 21.0 programme. Consistency of the age variable with the normal distribution was assessed using the Shapiro-Wilk test. Descriptive statistics are provided as median (range) for continuous variables and frequency and percentage for categorical variables. Between groups comparisons for categorical variables Pearson chi-square, Fisher’s exact test, and the Fisher Freeman Halton test were used; Kruskal-Wallis and Mann-Whitney U tests were used to compare continuous variables. Bonferroni correction used in pairwise comparisons. *P* < 0.05 was considered statistically significant.

## RESULTS

A total of 3,492 patients were included in study. The median age of the patients was 50.0 (age range: 10-97) years and 59.9% (*n* = 2093) were female. A total of 78.5% of the participants (n=2741) were never vaccinated, 13.4% (n=469) were irregularly vaccinated, and 8.1% (n= 282) received regular annual vaccination. The median age of the regularly vaccinated group was higher than that of the other groups (*p* < 0.001). Regular vaccination rates were higher in women (*p* = 0.005), married people (*p* < 0.001), those with national health insurance (*p* < 0.001), and non-smokers (*p* < 0.001) (Table-I).

**Table-I:** Relationship between influenza vaccination status and sociodemographic characteristics.

Variables	All	Never vaccinated (n=2741)	Annually vaccinated (n=282)	Irregularly vaccinated (n=469)	p-value
Age median (range)	50 (10-97)	47 (10-94)^a^	66.50 (25-97)^b^	59 (10-90)^c^	<0.001
Sex					
Male	1399 (40.1%)	1.066 (38.9%)^a^	137 (48.6%)^a^	196 (41.8%)^a^	0.005
Female	2093 (59.9%)	1.675 (61.1%)^b^	145 (51.4%)^b^	273 (58.2%)^a^	
Education level					
Less than high school	2280 (65.3%)	1.775 (64.7%)^a.b^	190 (67.3%)^a^	315 (67.1%)^a^	0.125
High school	751 (21.5%)	615 (22.4%)^b^	51 (18%)^a^	85 (18.1%)^a^	
University	461 (13.2%)	351 (12.8%)^a^	41 (14.5%)^a^	69 (14.7%)^a^	
Marital status					
Married	2833 (81.1%)	2.251 (82.1%)^a^	214 (75.8%)^a^	368 (78.4%)^a^	<0.001
Single	314 (9.0%)	282 (10.2%)^b^	4 (1.4%)^b^	28 (5.9%)^a^	
Divorced	345 (9.9%)	208 (7.5%)^c^	64 (22.7%)^c^	73 (15.5%)^b^	
Health insurance					
National	3012 (86.3%)	2.377 (86.8%)^a^	270 (95.7%)^a^	365 (77.9%)^a^	<0.001
Private	28 (0.8%)	23 (0.8%)^a^	2 (0.7%)^a.b^	3 (0.6%)^a.b^	
National + Private	451 (12.9%)	341 (12.4%)^a^	10 (3.5%)^b^	100 (21.3%)^b^	
Smoking					
Smoker	630 (18.1%)	528 (19.3%)^a^	31 (10.9%)^a^	71 (15.1%)^a^	<0.001
Ex-smoker	415 (11.9%)	296 (10.8%)^b^	44 (15.6%)^b^	75 (15.9%)^b^	
Never smoked	2436 (70%)	1.906 (69.8%)^c^	207 (73.4%)^b^	323 (68.8%)^a^	

Different superscripts indicate statistical difference between related groups.

Having risk factors such as “Previous influenza history (p<0.001)”, “being over 65 years old or staying in a home for the aged or nursing home (p<0.001)”, “being pregnant (p<0.001)”, “having chronic pulmonary and cardiovascular system disease, including asthma (p<0.001)”, were associated with regular vaccination (Table-II).

**Table-II:** Influenza risk factors and reported influenza vaccination status.

Variables	All	Never vaccinated n (%)	All	Annually vaccinated n (%)	All	Irregularly vaccinated n (%)	p-value
I had an influenza infection last year	2.043	1.039 (50.9%)^a^	206	119 (57.8%)^a^	396	152 (38.4%)^a^	<0.001
I am over 65 years old or staying in a home for the aged or nursing home	812	199 (24.5%)^b^	232	99 (42.7%)^b^	257	122 (47.5%)^b^	<0.001
I am pregnant	812	40 (4.9%)^b^	232	0 (0.0%)^b^	257	0 (0.0%)^b^	<0.001
I have chronic pulmonary and cardiovascular system disease, including asthma	812	436 (53.7%)^b^	232	125 (53.9%)^a^	257	102 (39.7%)^b^	<0.001
I have a chronic metabolic disease, including diabetes	812	261 (32.1%)	232	88 (37.9%)	257	83 (32.3%)	0.241
Those prescribed by all physicians, based on health reports of children and adolescents aged 6 months to 18 years who received long-term acetylsalicylic acid treatment, based on their health/pregnancy status.	812	5 (0.6%)	232	0 (0.0%)	257	3 (1.2%)	0.258

Different superscripts indicate statistical difference between related groups.

Regular vaccination rates were higher in the participants who gave correct answers to the knowledge questions (p<0.001). Regular vaccination rates were low in those who did not believe that the vaccine was beneficial (p<0.001), believe that the vaccine is harmful (p=0.031), and stated that they feared the side effects of the vaccine (p=0.021). The relation between vaccination status and patient’s knowledge, opinions and behaviour shown in Table-III.

**Table-III:** Patient’s knowledge, opinions, and reported behaviour and reported influenza vaccination status.

Variables	All	Never vaccinated n (%)	All	Annually vaccinated n (%)	All	Irregularly vaccinated n (%)	p-value
I don’t know about the vaccine	2.623	1.469 (50.0%)^b^	172	15 (8.7%)^b^	436	175 (40.1%)^b^	<0.001
I do not believe it is useful	2.623	521 (19.9%)^b^	172	8 (4.7%)^b^	436	90 (20.6%)^a^	<0.001
I want to get it done but I do not want to pay	2.623	425 (16.2%)^b^	172	144 (83.7%)^b^	436	130 (29.8%)^b^	<0.001
I think it’s harmful	2.623	109 (4.2%)^b^	172	2 (1.2%)^a^	436	10 (2.3%)^a^	0.031
I’m afraid of its side effects	2.623	270 (10.3%)^a^	172	9 (5.2%)^b^	436	56 (12.8%)^a^	0.021
School-age children should be vaccinated	2.612	919 (35.2%)^b^	272	142 (52.2%)^b^	450	161 (35.8%)^a^	<0.001
Collective workers should be vaccinated	2.612	735 (28.1%)^b^	272	116 (42.7%)^b^	450	144 (32.0%)^a^	<0.001
People over the age of 65 should be vaccinated	2.612	970 (37.1%)^b^	272	198 (72.8%)^b^	450	258 (57.3%)^b^	<0.001
People at risk should be vaccinated	2.612	1.436 (54.9%)^b^	272	209 (76.8%)^b^	450	286 (63.6%)^b^	<0.001
I don’t believe there is a need for vaccination	2.612	428 (16.4%)^b^	272	21 (7.7%)^b^	450	48 (10.7%)^b^	<0.001
I know that the influenza shot is covered by the government	2.712	615 (22.7%)^b^	278	261 (93.9%)^b^	463	280 (60.5%)^b^	<0.001
I would like to have regular vaccines if the influenza vaccine is given free of charge.	2.712	1.380 (50.5%)^b^	280	279 (99.6%)^b^	465	347 (74.6%)^b^	<0.001

Different superscripts indicate statistical difference between related groups.

Both regular and irregular vaccination rates were higher among participants in the risky group for influenza (p<0.001). Participants in the at-risk group had greater knowledge and more positive attitudes regarding the vaccine’s benefit (*p* < 0.001), the recommended vaccine population (*p* < 0.001), and the conditions under which health insurance would pay for the vaccine (*p* < 0.001) than did the low-risk participants. Participants in the at-risk group declared that if the vaccine were administered free of charge, they would get vaccinated regularly (*p* < 0.001). (Table-IV).

**Table-IV:** Patient’s knowledge, opinions, and reported behaviour and influenza risk factor status.

Variables	Risk/No		Risk/Yes		p-value

	All	n (%)	All	n (%)	
I had an influenza infection last year	1.627	821 (50.5%)	1.018	489 (48%)	0.225
I do not believe it is useful	2.060	451 (21.9%)	1.171	168 (14.4%)	<0.001
I want to get it done but do not want to pay	2.060	417 (20.2%)	1.171	282 (24.1%)	0.011
I think it is harmful	2.060	86 (4.2%)	1.171	35 (2.9%)	0.088
I’m afraid of its side effects	2.060	222 (10.8%)	1.171	113 (9.7%)	0.313
School-age children should be vaccinated	2.063	814 (39.5%)	1.271	408 (32.1%)	<0.001
Collective workers should be vaccinated	2.063	676 (32.8%)	1.271	319 (25.1%)	<0.001
People over the age of 65 should be vaccinated	2.063	802 (38.9%)	1.271	624 (49.1%)	<0.001
People at risk should be vaccinated	2.063	1.150 (55.7%)	1.271	781 (61.5%)	0.001
I don’t believe there is a need for vaccination	2.063	318 (15.4%)	1.271	179 (14.1%)	0.295
I know that the influenza shot is covered by the government	2.158	559 (25.9%)	1.295	597 (46.1%)	<0.001
I would like to have regular vaccines if the influenza vaccine is given free of charge.	2.179	1.107 (50.8%)	1.298	899 (69.3%)	<0.001
Influenza vaccination status					
Never vaccinated		1.929 (88%)		812 (62.4%)	<0.001
Annually vaccinated		50 (2.2%)		232 (17.8%)	
Irregularly vaccinated		212 (9.6%)		257 (19.7%)	

## DISCUSSION

This study showed tdhat a total of 78.5% of the participants were never vaccinated, 13.4% received vaccination irregularly, and 8.1% received regular annual vaccination. Older adult patients, non-smokers, women, those with health insurance, and those in at-risk group had higher regular vaccination rates. Furthermore, participants with a high level of knowledge about the influenza vaccine had higher vaccination rates. A negative opinion and incorrect and insufficient information deterred most people from getting vaccinated.

Two different studies conducted in Turkey have reported vaccination rates of 7.4% and 19%.^15^,^16^ In Sagor et al., it was determined that 63.3% of the population of Saudi Arabia had never received the influenza vaccine.^13^ In population-based studies in Lebanon and Jordan, vaccination rates were reported as 27.6% and 20.0%, respectively.^17^,^18^ A previous study examined influenza vaccination rates in European countries and reported that vaccination rates for 29 European countries were insufficient.^19^ The current and prior studies indicate that influenza vaccination rates are insufficient.

The current study showed that demographic characteristics such as sex, marital status, health insurance, and not smoking affected the decision to vaccinate. Some studies have indicated that sex can act as a barrier to vaccination.^20^-^22^ or as a promoter of vaccination.^21^-^24^ Other studies have found that marital status may exert an influence, whereby unmarried individuals were less likely to be vaccinated.^21^,^22^ The relationship between smoking and the influenza vaccine has been examined in very few articles. Two different studies reported no relationship between smoking and vaccination. Older age is a strong predictor of being vaccinated in different national contexts.^17^,^18^,^22^ The importance of influenza vaccination among older adults and the attitude of families and physicians towards vaccination in older adults may be important. Besides, in order to increase the vaccination rates against seasonal influenza, we can suggest that the vaccination costs should be reduced.

Bertoldo et al. reported that only 64.7% of participants knew that influenza can be prevented by vaccination and that patients with chronic diseases are likely to develop severe forms of influenza.^23^ Other studies showed that knowledge of the influenza vaccine was reported by 19.6% of persons in the USA^24^, 42% in France^19^, and 29.8% in Lebanon.^17^ In all these studies, the relationship between a high knowledge level and positive attitude toward vaccines were emphasized. Dardalas et al. reported that a low level of knowledge reduced vaccination rates by causing negative attitudes and behaviours.^22^ They suggested that healthcare professionals can play a key role in eliminating misconceptions and misinformation about not being vaccinated by providing information to the public. This could help achieve high vaccination rates.^22^ Our results revealed that regular vaccination was associated with participants who ‘thought that the vaccine was needed’, ‘thought vaccine was beneficial’, ‘thought he/she had sufficient knowledge about the influenza vaccine’, ‘knows the at-risk population for influenza’, and ‘knows who suffered from chronic disease’. Our results agree with studies that emphasized the positive effect of knowledge on vaccination rates.

In our sample, the frequently reported reasons for not vaccinating were fear of side effects, belief of not being at risk for influenza, belief that vaccines were harmful, and the vaccination fee. Our data showed similar results to other studies.^22^,^25^,^26^ Patient education can increase knowledge and reduce the barriers to vaccination. We believe that family physicians should devote more time to patient education to increase vaccination rates.

The current study demonstrated that we still cannot increase influenza vaccination rates. In addition to previous studies, our study showed that a high level of knowledge about the vaccine and the awareness of the need and the benefit of the vaccine can increase vaccination rates. The current study helps GPs understand that educating people about the benefits of vaccination and reducing negative opinions about it can ensure that more individuals are regularly vaccinated. We recommend that family physicians and GPs question the negative opinions of influenza vaccine and inform all patients about the benefits of vaccination.

### Limitations of the study:

The study’s primary limitation was that the data were obtained through questionnaire forms and may be subject to self-report bias. Participants’ opinions may have inhibited their provision of accurate information regarding their knowledge, opinions, and influenza vaccination rates. Since participants’ income levels were not questioned, the relationship between economic status and influenza vaccination could not be explored. Although the research was conducted by sampling across the country, participants which included patients who visited the primary health care institution, so that the study may not reflect the entire population.

## CONCLUSION

The current study results has indicated an insuffient rate of influenza vaccination in Turkish community. The results show that influenza vaccination rates increased among married people, women, those in a nursing home, those with chronic disease, and with increasing age. Regular vaccination was associated with high level of knowledge and positive attitudes of participants. It was determined that participants with lower risk factors had a lower rate of vaccination, but would regularly get the influenza vaccine if vaccines were free. Future studies should be focused on implementing educational interventions for all community by both general practitioners and specialists. Moreover, there is need for a approach to resolving the financial deficit in vaccination focused on health promotion and disease prevention

### Authors’ Contribution:

**OG, FEC, BY, IE, EHA** conceived, designed and did statistical analysis, responsible and accountable for the accuracy and integrity of the work & editing of manuscript.

**OG, FEC, BY** did data collection and manuscript writing.

**OG, FEC, BY, IE, EHA** did review and final approval of manuscript.
